# Operative Treatment of Intra-Articular Distal Radius Fractures With versus Without Arthroscopy: study protocol for a randomised controlled trial

**DOI:** 10.1186/s13063-017-2409-2

**Published:** 2018-02-02

**Authors:** Marjolein A. M. Mulders, Caroline A. Selles, Joost W. Colaris, Rolf W. Peters, Mark van Heijl, Berry I. Cleffken, Niels W. L. Schep

**Affiliations:** 1 Trauma Unit, Department of Surgery, Academic Medical Center, University of Amsterdam, P.O. Box 22660, 1100 DD Amsterdam, The Netherlands; 2 Department of Orthopaedic Surgery, Erasmus Medical Center, P.O. Box 2040, 3000 CA Rotterdam, The Netherlands; 3 Department of Surgery, Maasstad Hospital, P.O. Box 9100, 3007 AC Rotterdam, The Netherlands

**Keywords:** Distal radius fracture, Articular, Displaced, Wrist arthroscopy, Wrist function, PRWE, Ligamentous injuries, Randomised controlled trial

## Abstract

**Background:**

In the past several years, an increase in open reduction and internal fixation (ORIF) for intra-articular distal radius fractures has been observed. This technique leads to a quicker recovery of function compared to non-operative treatment. However, some patients continue to have a painful and stiff wrist postoperatively. Arthroscopically assisted removal of intra-articular fracture haematoma and debris may improve the functional outcomes following operative treatment of intra-articular distal radius fractures. The purpose of this randomised controlled trial is to determine the difference in functional outcome, assessed with the Patient-Rated Wrist Evaluation (PRWE) score, after ORIF with and without an additional wrist arthroscopy in adult patients with displaced complete articular distal radius fractures.

**Methods:**

In this multicentre trial, adult patients with a displaced complete articular distal radius fracture are randomised between ORIF with an additional wrist arthroscopy to remove fracture haematoma and debris (intervention group) and conventional fluoroscopic-assisted ORIF (control group). The primary outcome is functional outcome assessed with the PRWE score after three months. Secondary outcomes are wrist function assessed with the Disability of the Arm, Shoulder and Hand (DASH) score, postoperative pain, range of motion, grip strength, complications and cost-effectiveness. Additionally, in the intervention group, the quality of reduction, associated ligamentous injuries and cartilage damage will be assessed. A total of 50 patients will be included in this study.

**Discussion:**

Although ORIF of intra-articular distal radius fractures leads to a quicker resume of function compared to non-operative treatment, some patients continue to have a painful and stiff wrist postoperatively. We hypothesise that, due to the removal of fracture haematoma and debris by an additional arthroscopy, functional outcomes will be better compared to the non-arthroscopically treated group.

**Trial registration:**

ClinicalTrials.gov, NCT02660515. Registered on 13 January 2016.

**Electronic supplementary material:**

The online version of this article (doi:10.1186/s13063-017-2409-2) contains supplementary material, which is available to authorized users.

## Background

In the last decade, an increase in open reduction and internal fixation (ORIF) for distal radius fractures has been observed [1–3]. In particular, intra-articular distal radius fractures, which comprise almost 50% of all fractures [4], are increasingly being treated operatively. This technique leads to a quicker resume of function in the first three to six months compared to non-operative treatment [5, 6]. However, some patients continue to have a painful and stiff wrist postoperatively. Arthroscopically assisted removal of intra-articular fracture haematoma and debris may improve the functional outcomes following operative treatment of intra-articular distal radius fractures [7, 8]. Moreover, during arthroscopy the quality of the reduction [7, 9, 10] and the presence of associated ligamentous injuries can be assessed [11–14].

Lindau et al. already examined the frequency of associated chondral and ligament lesions with arthroscopy in 50 patients in 1997 [14]. They described 35 subchondral haematomas in 16 cases, and an incidence of chondral lesions of approximately 33%. These lesions may lead to the development of osteoarthritis in the long term [15]. Additionally, 98% of the patients had a ligamentous injury. However, they found no major instability in these patients and it is uncertain if these injuries will be clinically relevant in the long term [16].

Although, no advantage of arthroscopically guided reduction over conventional fluoroscopic-assisted reduction in regard to functional and radiographic outcomes was found [17], to our knowledge no studies have been carried out to further examine the use of arthroscopy after ORIF to remove fracture haematoma and debris on functional outcomes. We hypothesise that, due to the removal of fracture haematoma and debris, functional outcomes will be better compared to the non-arthroscopically treated group. Therefore, the purpose of this randomised controlled trial (RCT) is to determine the difference in functional outcome, assessed with the Patient-Rated Wrist Evaluation (PRWE) score, after ORIF with and without an additional wrist arthroscopy in adult patients with displaced complete articular distal radius fractures. Furthermore, we aim to determine the difference in functional outcomes with the Disability of the Arm, Shoulder and Hand (DASH) score, postoperative pain, range of motion (ROM), grip strength, complications, and cost-effectiveness. Additionally, the quality of reduction, associated ligamentous injuries and cartilage damage will be assessed by arthroscopy.

## Methods/Design

### Study objectives

The primary objective is to determine the difference in functional outcome of ORIF with or without an additional arthroscopy to remove the fracture haematoma and debris in adult patients with displaced complete articular distal radius fractures (AO/OTA type C).

The secondary objectives are to assess if additional wrist arthroscopy leads to less postoperative pain, a better ROM and grip strength, and fewer complications. Additionally, cost-effectiveness for both treatments is determined. Moreover, for patients undergoing additional wrist arthroscopy, the quality of reduction, associated ligamentous injuries and cartilage damage will be assessed.

### Study design

The RADAR (Operative Treatment of Intra-Articular Distal Radius Fractures With versus Without Arthroscopy) trial is designed as a multicentre RCT, with a 1:1 allocation ratio and a superiority framework. Patients are randomised between ORIF with an additional wrist arthroscopy to remove fracture haematoma and debris (intervention group) and conventional fluoroscopic-assisted ORIF (control group). A total of three centres in the Netherlands are involved in recruiting patients (Additional file 1).

The design of the trial is compliant with the Standard Protocol Items: Recommendations for Interventional Trials [18] (Additional file 2).

### Study population

The study population will consist of all adult patients who are diagnosed with a complete articular distal radius fracture (AO/OTA type C) where the treating surgeon deems ORIF necessary. Independent radiologists will assess and classify complete articular distal radius fracture based on radiography according to the AO/OTA classification of fractures. All patients undergo a computed tomography (CT) scan of the wrist. This is standard care in decision-making and planning for surgery [19].

### Inclusion criteria

- Patients aged 18 years and older
- Displaced complete articular distal radius fracture (AO/OTA type C) as classified on lateral, posterior-anterior, and lateral carporadial radiographs by a radiologist or trauma surgeon, requiring ORIF. An additional dorsal approach is allowed only when the dorsal capsule is not opened and thus leaving the radiocarpal joint untreated.
- Inacceptable alignment on radiograph defined, according to the Dutch National Guidelines [19], as:
  - ○ radial inclination < 15°;
  - ○ radial length (distance between lateral most radial tip and ulnar surface) ≤ 5 mm;
  - ○ volar angulation ≥ 20° or dorsal angulation ≥ 15°;
  - ○ articular step-off or gap ≥ 2 mm. A gap is defined as loss of articular congruity of the distal radius parallel to the articular surface and a step-off perpendicular to the articular surface [20].

### Exclusion criteria

- Dorsal plate fixation in case the radiocarpal joint needs to be opened
- Multiple trauma patients (Injury Severity Score (ISS) ≥ 16)
- Open distal radius fractures
- Other fractures in the ipsilateral extremity (except for a fracture of the ulnar styloid process)
- Fracture of the contralateral wrist (distal radius, distal ulna or one of the carpal bones)
- Patients with impaired wrist function before injury due to arthrosis, rheumatoid arthritis, neurological disorders or malunion of the upper limb or patients suffering from disorders of bone metabolism other than osteoporosis (i.e. Paget’s disease, renal osteodystrophy, osteomalacia) or connective tissue disease or (joint) hyperflexibility disorders such as Marfan’s or Ehler Danlos
- Patients with insufficient comprehension of the Dutch language to understand the study information and informed consent process, the rehabilitation program and other treatment information as judged by the attending physician

### Interventions

All patients will be treated by a certified (orthopaedic) trauma surgeon with experience in ORIF of distal radius fractures and wrist arthroscopy. In both groups ORIF of the distal radius fracture will be similar. The intervention group will be treated with wrist arthroscopy following ORIF. A delay of at least five days before performing arthroscopy is mandatory to enable visualisation due to the organisation of the hematoma. The operation has to be performed within three weeks after the initial trauma.

Antibiotic prophylaxis (Cefazoline, 1000 mg i.v.) is given preoperatively, according to the current standard. The volar approach according to Henry will be used [21]. This entails an incision between the radial artery and the tendon of the flexor carpi radialis. The pronator quadratus muscle from will be detached from its distal and lateral side and lifted for optimal exposure to the fracture site. After the fracture site is revealed, the fracture will be debrided, reduced and fixated with an appropriate volar locking plate. The type and brand of the plate are at the discretion of the treating surgeon. When a dorsal approach is deemed necessary the distal radius will be approached through the third dorsal extensor tendon compartment, without opening the dorsal capsule. Fluoroscopic images are obtained to evaluate the quality of articular reduction. Wrist arthroscopy will be performed when the treating surgeon is satisfied with the result of the ORIF.

**Fig. 1:**
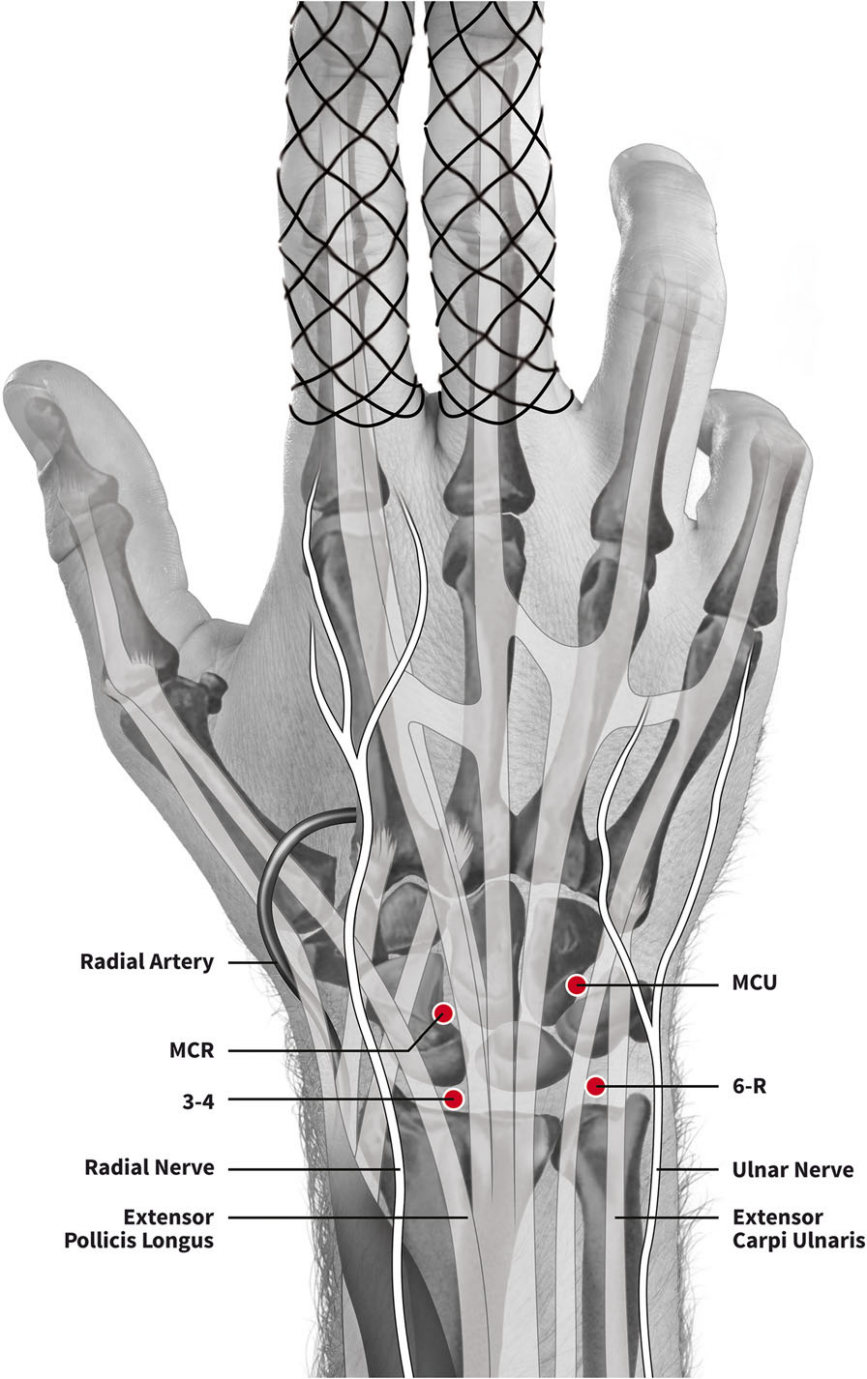
View of wrist arthroscopy portals

During wrist arthroscopy, the forearm will be positioned upright and in neutral position, the elbow flexed by 90° and axial traction of 4–6 kg will be performed. Four portals are created dorsally by superficial stab incisions and blunt preparation through the joint capsule; one midcarpal radial (MCR) and ulnar (MCU) portal and one radiocarpal 3-4 and 6-R portal (Fig. 1). Portals may be changed to improve visualisation. A shaver or mini grasper is used for removal of fracture haematoma and osteocartilaginous debris. Cartilage damage will be graded using the Outerbridge classification system [22] (Additional file 3). With the 1-mm hook probe, assessment of the quality of reduction and ligamentous injuries will be performed. Step-off and gaps will be measured with a calibrated 1-mm probe at the point of maximum displacement and recorded. The trampoline and hook test are performed to demonstrate a triangular fibrocartilage complex (TFCC) tear. TFCC tears will be classified according to Palmer [23] (Additional file 4). All scapholunate ligament injuries will be noted and graded according to the Geissler classification [11] (Additional file 5). The same classification will be applied for lunotriquetral injuries. Wound closure will be performed using standard techniques. All patients will receive a pressure bandage for 24–48 h.

For both the intervention and the control group, patients are allowed to start exercising immediately after the operation. Exercises include pronation and supination, flexion and extension, and ulnar and radial deviation of the wrist. Patients are instructed to use the affected extremity as far as pain allows. However, only non-weight-bearing practice is allowed for the first six weeks. Rehabilitation with the assistance of a physiotherapist is recommended at the discretion of the patient and treating surgeon.

All interventions are performed according to predefined Standard Operating Procedures (SOPs). Individuals can leave the study at any time for any reason if they wish to do so without any consequences. The investigator can decide to withdraw a participant from the study for urgent medical reasons.

### Outcomes

#### Primary outcome

**Fig. 2:**
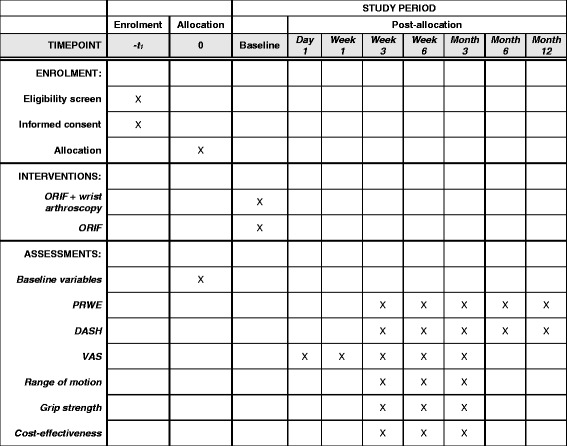
Follow-up visits

The primary outcome of this study is wrist pain and disability expressed as change on the PRWE score after three months. In addition, the PRWE questionnaire will be completed after three and six weeks, and six and 12 months of follow-up (Fig. 2). The PRWE is a validated tool for assessing functional outcome in patients with distal radius fractures [24, 25]. The PRWE is a 15-item questionnaire which measures wrist pain and disability in activities of daily living on a scale of 0–10. Although the PRWE consists of three subscales (pain, function and cosmetics), the PRWE results in a single score [26]. The highest score, indicating severe impairment, is 100 and the best score, indicating no impairment, is zero. The Dutch version has been structurally validated [26]. The PRWE score will be expressed as a final value at each of the follow-up moments.

#### Secondary outcomes

- Wrist function, disability and pain as measured with the DASH score, at three and six weeks and three, six and 12 months of follow-up (Fig. 2). The DASH questionnaire is a 30-item, self-report questionnaire which measures physical function and symptoms in patients with any or several musculoskeletal disorders of the upper limb, including the distal radius [27–29]. The DASH questionnaire tests the degree of difficulty in performing a variety of physical activities because of arm, shoulder or hand problems (six items), the severity of pain, tingling (two items), as well as the effect of the upper limb problem on social activities, work and sleep (three items). The highest score is 100, indicating severe disability and pain; the lowest score is zero, indicating no disability and pain. The Dutch version of the DASH questionnaire has been validated and has shown to be a reliable and valid instrument [30]. The DASH score will be expressed as a final value at each of the follow-up moments.
- Postoperative pain as indicated on a Visual Analogue Scale (VAS), where zero means no pain and ten the worst pain possible. Patients will be asked to give an estimation of their pain and the type and quantity of pain medication taken postoperatively at one day, one week, three weeks, six weeks and three months (Fig. 2). The VAS pain score will be expressed as the final value at each of the follow-up moments.
- ROM of the wrist measured on both the injured as well as the uninjured wrist with a handheld goniometer. Measurements of ROM include ulnar and radial deviation, pronation and supination, and flexion and extension of the wrist. ROM is measured at three weeks, six weeks and three months, and will be expressed as both a final value and as a percentage of the uninjured side (Fig. 2).
- Prehensile grip strength as a percentage of the uninjured wrist. Grip strength will be measured on both sides with a Baseline dynamometer (White Plains, NY, USA) with the arm of the patient to the side and the elbow at 90° flexion. Grip strength will be calculated as the mean of three measurements and expressed as a final value and as a percentage of the uninjured side. Grip strength is measured at three weeks, six weeks and three months (Fig. 2).
- Complications, such as superficial or deep infection divided by the criteria of the US Centers for Disease Control and Prevention [31], tendinitis or rupture of one of the flexor or extensor tendons, carpal tunnel syndrome, compartment syndrome, complex regional pain syndrome (CRPS) type 1 according to the Veldman [32] and the Budapest criteria [33, 34], and hardware-related complications will be recorded.
- Cost-effectiveness and cost-utility of ORIF with and without an arthroscopic-assisted procedure from a societal perspective, measured with an economic evaluation questionnaire at three weeks, six weeks and three months follow-up. The economic evaluation questionnaire is based on the EQ-5D and the Standard Form Health and Labour questionnaire. The EQ-5D will be used to measure quality-adjusted life years (QALY). Since this analysis is from a societal perspective, direct healthcare costs, direct non-healthcare costs and indirect costs due to the operative treated distal radius fracture will be considered (Table 1). A more detailed description of the economic analysis can be found in the protocol of the VIPAR trial [35]. The cost-effectiveness is determined at three weeks, six weeks and three months (Fig. 2).

**Table 1:** Costs included in the economic evaluation

Direct healthcare costs
Open reduction and internal fixation
Additional cost of wrist arthroscopy
Follow-up visits medical specialist
Additional visits to healthcare professional
Prescribed medication
Professional home care
Treatment and follow-up of complications
Physical therapy
Direct non-healthcare costs
Travel expenses to and from the hospital
Over the counter medication
Care provided by family or paid help
Assistive devices
Indirect costs
Absenteeism from paid labour (per day)
- In the intervention group the quality of reduction, associated ligamentous injuries and cartilage damage will be assessed. Ligamentous injuries are divided in TFCC injuries, classified according to the Palmer classification [23], and scapholunate ligament and lunotriquetral injuries, graded according to the Geissler classification [11].

### Randomisation

All consecutive adult patients who are diagnosed with a displaced complete articular distal radius fracture (AO/OTA type C) and scheduled for ORIF will be invited to participate in this study if they meet the inclusion and exclusion criteria. Informed consent will be obtained at the outpatient clinic before the operation. Randomisation will be performed by means of a computerised randomisation procedure, using Castor®, which is an online secure randomisation service. Allocation concealment will be ensured until patients have been randomised, which takes place after baseline characteristics have been obtained. The sequence of allocation is concealed until trial completion. To avoid imbalances between treatment groups, patients will be randomised in two strata according to age: 18–65 years and ≥ 65 years using a mixed block randomisation with blocks of four, six and eight patients. The order of the block sizes is unknown to the researchers, who therefore remain blinded to the allocation of the next individual throughout the whole study.

### Blinding

Since the treatment allocation involves a surgical procedure and therefore the surgical incision and portal entrees will be visible for both physician and patient, randomisation status will not be blinded.

### Sample size calculation

The sample size calculation is based on our primary outcome, the PRWE score. We choose the PRWE score at three months as our primary outcome, since we expect patients to profit most from additional wrist arthroscopy within three months after the initial trauma. After this point, the haematoma has dissolved without intervention. The mean PRWE score after a distal radius fracture after three months of follow-up in adult patients is 28 with a standard deviation of 21.3 [36]. This PRWE score was measured in a population in which 38% of patients suffered from a complete articular distal radius fracture (AO/OTA type C fracture). Although this cohort of patients is not fully comparable to our cohort of patients, it is the data which most closely resembles our study population. We chose an effect size of 18 points on the PRWE score at three months, since we expect the greatest difference in PRWE score between both groups at three months of follow-up. The minimally clinically important difference is set at 11.5, therefore every difference > 11.5 is clinically meaningful [37]. Therefore, at α = 0.05% and a power of 80%, we would require 46 patients in total and 23 per treatment arm. For safety measures and with an expected loss to follow-up of 5%, 25 patients in each arm will be included. In a separate study conducted in the Netherlands by our research group, a prevalence of AO/OTA type C distal radius fractures of approximately 25% was found [4]. Therefore, we estimate to include and follow-up all 50 patients in a maximum of 1.5 years.

### Data analysis

All patients will be analysed according to the intention-to-treat protocol. General descriptive statistics on patient characteristic at baseline will be performed including factors such as gender and age, and presented as percentages (categorical variables) or means and standard deviation (SD) (continuous variables), whichever is applicable. Normality will be determined by visually inspecting the plotted data distribution in a histogram. Differences between the two groups in the primary outcome, the PRWE score, will be analysed using an analysis of covariance (ANCOVA), corrected for age. The same applies for the DASH score at the different follow-up moments. The secondary outcomes—pain (VAS), ROM and grip strength—will be analysed using a linear mixed model. The best covariance structure for each linear mixed model is determined using the smallest Akaike information criterion (AIC). The VAS pain score will be corrected for painkiller use. Differences in complication rates between the two treatment groups will be analysed using the Chi-square test or Fisher’s exact test (in case the expected incidence is less than five). Subgroup analyses will be performed on gender and age. Multiple imputation will be used in case of > 10% missing data.

### Data management and monitoring

All follow-up moments are part of the regular outpatient clinic appointments. Data of patients lost to follow-up will be analysed until the last follow-up appointment. Data will be stored in two separate files. One dataset will contain coded patient information, based on an unambiguous identification code, and a second set of medical history linked to these codes. The coordinating investigator safeguards the key to the code. The same applies for all screened patients. Data are entered in Castor®. All entered data and changes are saved; a list is maintained of all individuals who are authorised to make data changes. A reason is always indicated when changes are made to the data. All data are adequately backed up and can be retrieved form the archive. All researchers involved in the study will have access to all data collected. Data will be stored and kept for 15 years according standard guidelines.

The Institutional Review Board waived the need for a data monitoring committee, since both treatment modalities are part of standard care. An audit is performed half way during the trial.

### Protocol amendments

For any modifications of the study protocol (29 December 2016; version 6) that may impact the study, approval will be obtained from the Institutional Review Board before implementation. Protocol modifications are communicated to relevant parties by letter.

### Adverse events

All adverse events will be described in the patient file during consult at any of the follow-up visits or any other moment if indicated or requested by the patient. This includes wound infection, complex regional pain syndrome, compartment syndrome and any neurovascular or tendon damage. Complex regional pain syndrome will be classified according to the ‘Budapest Criteria’ created and validated by the Budapest consensus group [33, 34].

All serious adverse events (SAE) are reported to the accredited medical ethics board that approved the protocol, within 15 days after the sponsor has first knowledge of the serious adverse reactions. Arthroscopic-related complications which require a readmission or reoperation are listed in a periodic overview.

SAEs that result in death or are life-threatening should be reported expeditiously. The expedited reporting will occur not later than seven days after the responsible investigator has first knowledge of the adverse reaction. This is for a preliminary report with another eight days for completion of the report.

All adverse events will be followed until they have abated or until a stable situation has been reached. Depending on the event, follow-up may require additional tests or medical procedures as indicated, and/or referral to the general physician or a medical specialist.

### Ethics

This study will be conducted according to the principles of the Declaration of Helsinki (64th WMA General Assembly, Fortaleza, Brazil, October 2013) and in accordance with the Medical Research Involving Human Subjects Act (WMO) and ‘Good Clinical Practice’ guidelines. Insurance was set up for compensation for the study participants who suffer from potential harm.

### Dissemination policy

The results of this study will be submitted for publication in a peer-reviewed journal. The criteria for authorship will follow the guidelines established by the International Committee of Medical Journal Editors.

## Discussion

Randomisation status will not be blinded, since the treatment allocation involves a surgical procedure and therefore the surgical incision and the portal entrees are visible for both physician and patient.

### Trial status

This trial has finished recruiting patients.

## Additional files

Additional file 1: Participating centres. (DOC 23 kb)

Additional file 2: SPIRIT 2013 checklist. Standard Protocol Items: Recommendations for Interventional Trials. (DOC 123 kb)

Additional file 3: Outerbridge classification for cartilage damage. (PDF 191 kb)

Additional file 4: Palmer classification for TFCC acute traumatic tears. (PDF 118 kb)

Additional file 5: Geissler classification for SL and LT tears. (PDF 106 kb)

## Abbreviations

AO/OTA: Arbeitsgemeinshaft für Osteosynthesefragen/Orthopedic Trauma Association
CT: Computed tomography
DASH: Disability of the Arm, Shoulder and Hand
ISS: Injury Severity Score
MCR: Midcarpal radial
MCU: Midcarpal ulnar
ORIF: Open reduction and internal fixation
PA: Posterior-anterior
PRWE: Patient-Rated Wrist Evaluation
ROM: Range of motion
SAE: Serious adverse event
SD: Standard deviation
VAS: Visual Analogue Scale
