# Supplementary material for: Operative Treatment of Intra-Articular Distal Radius Fractures With versus Without Arthroscopy: study protocol for a randomised controlled trial
Source: Trials. 2018 Feb 2;19:84. doi: 10.1186/s13063-017-2409-2 (PMC5797370; doi:10.1186/s13063-017-2409-2)
Supplement: Supplementary file 1 — Participating centres. (DOC 23 kb) [file 13063_2017_2409_MOESM1_ESM.doc]

Additional file 1. Participating centres

1. Maasstad Hospital, Rotterdam, The Netherlands; coordinating centre. Is responsible for compiling a steering committee and trial management committee. Will provide a principal investigator, and at least one member for the steering committee and for the trial management committee.
2. Erasmus Medical Center, Rotterdam, The Netherlands; participating centre. Will provide a lead investigator, and one member for the steering committee.
3. Academic Medical Center, Amsterdam, The Netherlands; participating centre. Will provide a lead investigator, and one member for the steering committee.
