# Supplementary material for: Operative Treatment of Intra-Articular Distal Radius Fractures With versus Without Arthroscopy: study protocol for a randomised controlled trial
Source: Trials. 2018 Feb 2;19:84. doi: 10.1186/s13063-017-2409-2 (PMC5797370; doi:10.1186/s13063-017-2409-2)

Additional file 3. Outerbridge classification for cartilage damage

- Grade 0: normal cartilage
- Grade I: cartilage with softening and swelling
- Grade II: partial-thickness defect with fissures on the surface that do not reacht subchondral bone or exceed 1.5 cm in diameter
- Grade III: fissuring to the level of subchondral bone in an area with a diatmeter more than 1.5 cm
- Grade IV: exposed subchondral bone

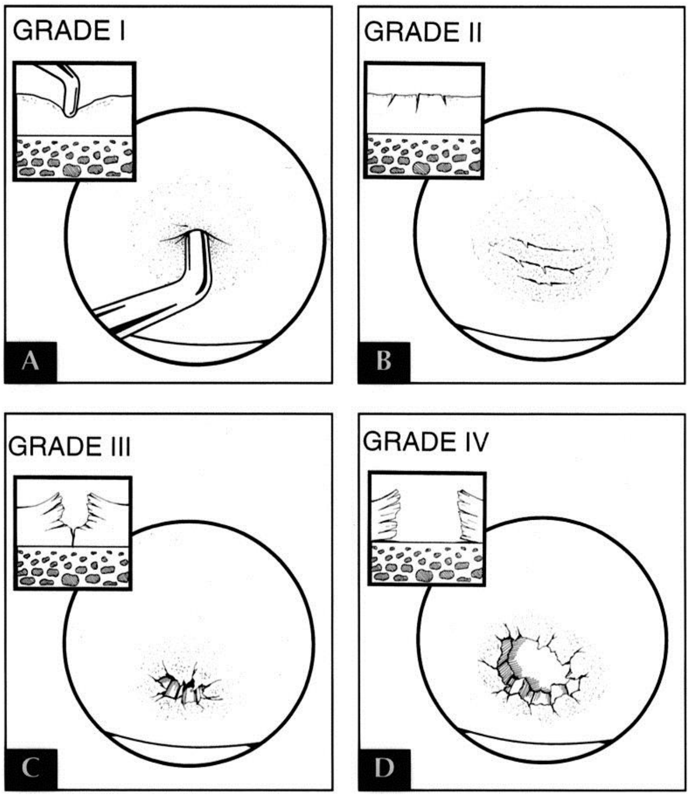

Supplement: Supplementary file 3 — Outerbridge classification for cartilage damage. (PDF 191 kb) [file 13063_2017_2409_MOESM3_ESM.pdf]
