# Supplementary material for: Operative Treatment of Intra-Articular Distal Radius Fractures With versus Without Arthroscopy: study protocol for a randomised controlled trial
Source: Trials. 2018 Feb 2;19:84. doi: 10.1186/s13063-017-2409-2 (PMC5797370; doi:10.1186/s13063-017-2409-2)

Additional file 4. Palmer classification for TFCC acute traumatic tears

- A: central perforation
- B: Ulnar avulsion with or without distal ulnar fracture (may involve the proximal or distal lamina (foveal and styloid attachment, respectively) or both)
- C: Distal avulsion
- D: Radial avulsion with or without sigmoid notch fracture

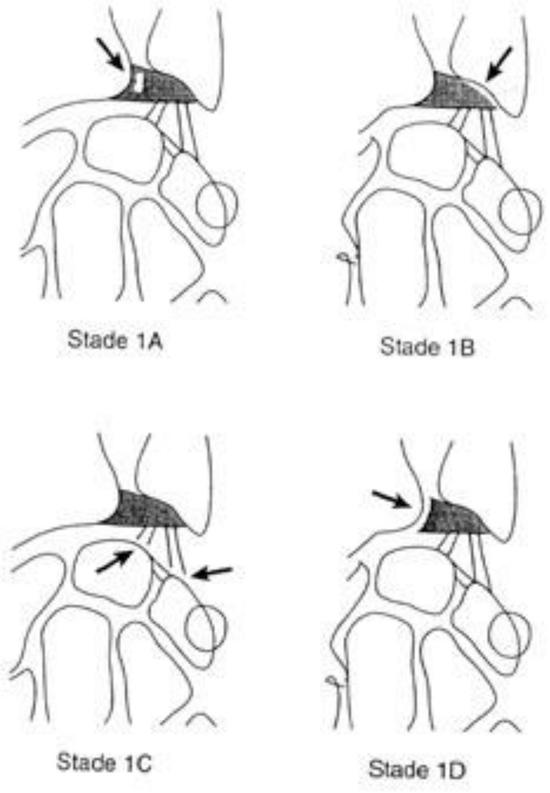

Supplement: Supplementary file 4 — Palmer classification for TFCC acute traumatic tears. (PDF 118 kb) [file 13063_2017_2409_MOESM4_ESM.pdf]
