# Supplementary material for: Operative Treatment of Intra-Articular Distal Radius Fractures With versus Without Arthroscopy: study protocol for a randomised controlled trial
Source: Trials. 2018 Feb 2;19:84. doi: 10.1186/s13063-017-2409-2 (PMC5797370; doi:10.1186/s13063-017-2409-2)
Supplement: Supplementary file 5 — Geissler classification for SL and LT tears. (PDF 106 kb) [file 13063_2017_2409_MOESM5_ESM.pdf]

Additional file 5. Geissler classification for SL and LT tears

- I: Attenuation and/or hemorrhage of the interosseous ligament as observed from the radiocarpal joint. No incongruence of carpal alignment in midcarpal space
- II: Attenuation and/or hemorrhage of the interosseous ligament, as observed from the radiocarpal joint. Incongruence and/or step-off, as observed from the midcarpal space. A slight gap (less than the width of a probe) between the carpals may be present.C: Distal avulsion
- III: Incongruence and/or step-off of the carpal alignment are observed in both the radiocarpal and midcarpal space. The probe may be passed through a gap between the carpals
- IV: Incongruence and/or step-off of the carpal alignment are observed in both the radiocarpal and midcarpal space. Gross instability with manipulation is noted. A 2.7-mm arthroscope may be passed through the gap between the carpals.
